# Retinotopic patterns of background connectivity between V1 and fronto-parietal cortex are modulated by task demands

**DOI:** 10.3389/fnhum.2015.00338

**Published:** 2015-06-08

**Authors:** Joseph C. Griffis, Abdurahman S. Elkhetali, Wesley K. Burge, Richard H. Chen, Kristina M. Visscher

**Affiliations:** ^1^Department of Psychology, University of Alabama at BirminghamBirmingham, AL, USA; ^2^Department of Neurobiology, University of Alabama at BirminghamBirmingham, AL, USA

**Keywords:** fMRI, background connectivity, eccentricity, visual cortex, V1, attention, cognitive control, fronto-parietal network

## Abstract

Attention facilitates the processing of task-relevant visual information and suppresses interference from task-irrelevant information. Modulations of neural activity in visual cortex depend on attention, and likely result from signals originating in fronto-parietal and cingulo-opercular regions of cortex. Here, we tested the hypothesis that attentional facilitation of visual processing is accomplished in part by changes in how brain networks involved in attentional control interact with sectors of V1 that represent different retinal eccentricities. We measured the strength of background connectivity between fronto-parietal and cingulo-opercular regions with different eccentricity sectors in V1 using functional MRI data that were collected while participants performed tasks involving attention to either a centrally presented visual stimulus or a simultaneously presented auditory stimulus. We found that when the visual stimulus was attended, background connectivity between V1 and the left frontal eye fields (FEF), left intraparietal sulcus (IPS), and right IPS varied strongly across different eccentricity sectors in V1 so that foveal sectors were more strongly connected than peripheral sectors. This retinotopic gradient was weaker when the visual stimulus was ignored, indicating that it was driven by attentional effects. Greater task-driven differences between foveal and peripheral sectors in background connectivity to these regions were associated with better performance on the visual task and faster response times on correct trials. These findings are consistent with the notion that attention drives the configuration of task-specific functional pathways that enable the prioritized processing of task-relevant visual information, and show that the prioritization of visual information by attentional processes may be encoded in the retinotopic gradient of connectivty between V1 and fronto-parietal regions.

## Introduction

Cognitive control influences visual processing at the earliest stages of the visual system. Primary visual cortex, also known as V1, is the earliest cortical area devoted to the processing of visual information (Felleman and Van Essen, 1991). V1 is organized as a retinotopic map of visual space where anterior parts of cortex represent peripheral vision and posterior parts of cortex represent central vision (Fox et al., 1987; Engel et al., 1997; Hadjikhani and Tootell, 2000). While V1 plays a relatively basic role in processing visual information, neural activity in V1 is modulated by higher order cognitive processes such as attention (Watanabe et al., 1998, 2011; Kelly et al., 2008; Poghosyan and Ioannides, 2008). For example, cues that precede attended visual stimuli result in retinotopically specific changes in V1 activity that likely result from top-down preparatory signaling (for review, see Carrasco, 2011). Accordingly, top-down modulations of neural activity in V1 and other early visual areas are thought to facilitate visual processing by enhancing the responses of neurons that correspond to task-relevant information (Tootell et al., 1998; Kastner et al., 1999; Somers et al., 1999; Giesbrecht et al., 2006; Munneke et al., 2008; Sylvester et al., 2009) and suppressing the responses of neurons that correspond to task-irrelevant information (Worden et al., 2000; Kelly et al., 2006; Ruff and Driver, 2006; Thut et al., 2006; Rihs et al., 2007; Sylvester et al., 2008).

The processing of visual information is also influenced by ongoing, stimulus-independent fluctuations in the baseline level of neural activity in early visual areas. Often referred to as “ongoing” or “background” neural activity, these fluctuations are thought to reflect dynamic changes in the cortical state of early visual areas that lead to variable evoked neural responses (Arieli et al., 1996; Tsodyks et al., 1999; Ringach, 2009). Similar to stimulus-evoked responses, background neural activity in early visual areas is modulated by top-down cognitive processes such as attention (Chawla et al., 1999; Silver et al., 2007; Sylvester et al., 2009; Cardoso et al., 2012; Elkhetali et al., 2015). For example, baseline activity in V4 and V5 is modulated during attention to color and motion, and these shifts in baseline activity likely serve to influence the population responses to incoming visual stimulation (Chawla et al., 1999). Similarly, a recent study by our lab found that tasks requiring prolonged attention to a visual stimulus result in shifts in the baseline level of neural activity in early visual areas including V1 that are independent of stimulus factors and are interpreted as aiding in the maintenance of a stable task set (Elkhetali et al., 2015).

There is also evidence that background neural activity in early visual areas might reflect ongoing interactions with other parts of the brain. For example, the correlation of background neural activity between early visual areas and higher-level visual areas that are specialized for processing certain types of visual information is modulated depending on what types of visual information are attended (Al-Aidroos et al., 2012; Norman-Haignere et al., 2012). The correlation in the background neural activity of two different areas is often referred to as background connectivity. It has been proposed that changes in the background connectivity of early visual areas reflect neural dynamics underlying the transfer of visual information. This interpretation is supported by evidence that spontaneous changes in visual perception relate to changes in the background activity in sectors of V1 and other early visual areas that correspond to specific retinotopic locations (Donner et al., 2013). Spontaneous changes in background activity also correlate with variability in performance on visual attention tasks (Haynes et al., 2005; Schölvinck et al., 2012). Thus, it is possible that task-driven changes in the background neural activity of early visual areas reflect the configuration of task-specific cortical states that arise partly from dynamic “background” interactions between early visual areas and brain areas involved in the top-down control of visual processing.

The fronto-parietal and cingulo-opercular networks are two large-scale brain networks that have been shown to play distinct but complementary roles in the top-down control of sensory processing (Dosenbach et al., 2007). The fronto-parietal network, in particular, has been strongly implicated in the top-down control of visuo-spatial attention and is considered to be a primary source of attention-driven changes in the neural activity of early visual areas (Hopfinger et al., 2000; Giesbrecht et al., 2003; Grent-’t-Jong and Woldorff, 2007; Kastner et al., 2007; Bressler et al., 2008; Szczepanski et al., 2010, 2014; Zanto et al., 2010, 2011; McMains and Kastner, 2011; Simpson et al., 2011; Greenberg et al., 2012; Nelissen et al., 2013). Further, some regions in frontal and parietal cortex encode topographic maps of visual space that reflect the prioritization of visual locations (Kastner et al., 2007; Arcizet et al., 2011; Jerde et al., 2012; Mirpour and Bisley, 2012; Ptak, 2012; Klink et al., 2014), indicating that visuo-spatial information is salient to processing in these higher order brain regions. Thus, it might be expected that these regions may modulate neural activity in specific eccentricity sectors of early visual areas to prioritize the processing of relevant visual information. Such an explanation is supported by studies using transcranial magnetic stimulation (TMS) to investigate the effective connectivity of two key nodes of the fronto-parietal network: the frontal eye fields (FEF) and intraparietal sulcus (IPS). Evidence from these studies indicates that stimulation of these regions can modulate background activity in early visual cortex in a retinotopically specific fashion (Ruff et al., 2006, 2007, 2009), although it is not clear if the effects of TMS are analogous to the effects of task-dependent attentional deployment.

The cingulo-opercular network includes the dorsal anterior cingulate cortex, and the anterior insula/frontal operculum, among other regions. This network is primarily involved in more general state-related attentional processes such as the initiation and maintenance of task sets (Dosenbach et al., 2007). While the precise role of this network in the top-down control of visual processing is less clear, there is evidence that tasks involving visuo-spatial attention lead to increased background connectivity between V1 and the anterior insula (Ebisch et al., 2013). It is thus possible that task-driven changes in neural activity in V1 associated with task set maintenance (e.g., Elkhetali et al., 2015) arise due to interactions between V1 and this network.

We hypothesized that the strength of background connectivity between different eccentricity sectors in V1 and regions responsible for the deployment of visual attention would depend on whether a visual stimulus was attended or ignored. To test this hypothesis, we used BOLD fMRI to measure background connectivity between V1 and nodes of the fronto-parietal and cingulo-opercular networks during task conditions that required attention to either a centrally presented visual stimulus or a simultaneously presented auditory stimulus. Because identical stimulation was used for each condition, this task design enabled us to measure changes in background connectivity that could not be attributed to between-condition differences in stimulus parameters or general arousal. Because the visual stimulus was presented centrally, we expected that foveal V1 would show increased connectivity with control network nodes during the visual condition compared to the auditory condition, and that peripheral V1 would either show no difference in connectivity between conditions or would show reduced connectivity during the visual condition compared to the auditory condition. Further, we expected that if task-driven changes in background connectivity reflected the action of top-down processes that facilitated visual processing, then participants with larger between-condition differences in connectivity between control network nodes and task-relevant (foveal) vs. task-irrelevant (peripheral) eccentricity sectors would also perform better on the visual task.

## Methods

### Participants

All participants were recruited through campus-wide advertisements. Recruitment policies adhered to ethical standards as set and reviewed by the IRB at the University of Alabama at Birmingham. All participants provided a written consent prior to admission to the study. Data were collected from 20 healthy right-handed participants aged 19–29 (8 males, 12 females; mean age = 26) with normal hearing and normal or corrected-to-normal vision. For *a priori* analyses incorporating behavior, we wanted to ensure that the behavioral analyses accurately reflected the relationship between background connectivity and task performance. Therefore, data from five subjects were excluded because of hardware issues that resulted in behavioral data not being collected for greater than 10% of trials on the visual task (behavioral data for 40% of trials was missing for two subjects, behavioral data for 30% of trials was missing for two subjects, and behavioral data for 20% of trials was missing for one subject). Thus, data from the remaining 15 subjects were used for all background connectivity and behavioral analyses (seven males, eight females; mean age = 26).

### Stimulus Parameters and Task Design

Functional MRI and behavioral data were collected while participants performed an attention demanding discrimination task that had conditions with auditory and visual target modalities. Full descriptions of the stimulus parameters have been previously published (Elkhetali et al., 2015), and will be briefly described here. The visual stimuli consisted of gray-scale horizontal gratings, often called “Gabor patches”, that were presented centrally and varied in luminance sinusoidally over space. The Gaussian window defining the contrast of the bars in the Gabor patch had a standard deviation of 2.71° visual angle. The visibility of the visual stimuli decreased with the distance from the center of the screen (the area of highest contrast). At 5.4° visual angle, the image had zero contrast, and thus the visible portion of the Gabor ended between 2.7 and 5.4° eccentricity. Auditory stimuli varied sinusoidally over time and in tone and have previously been described as “ripple sounds” (Shamma, 2001).

A detailed description of the task design has been previously published (Elkhetali et al., 2015), and will be briefly described here. Trials were presented using a mixed blocked/event-related design (Visscher et al., 2003) that alternated between task blocks and rest blocks. Each scan session consisted of eight runs that contained five task blocks consisting of eight trials separated by a jittered inter-trial interval ranging from 4–14 s. Task blocks lasted 70 s each, and each task block was flanked by a rest block that lasted 24 s. A small white fixation cross was presented centrally at the beginning of each run and during each rest block, and participants were instructed to maintain fixation throughout the duration of the block. For each task block, the target modality (visual or auditory) was indicated by the presentation of a colored cue that appeared at the location of the fixation cross. The presentation of a yellow cross-hair indicated that participants should attend the auditory stimulus and ignore the visual stimulus, while the presentation of a blue cross-hair indicated that participants should attend the visual stimulus and ignore the auditory stimulus.

For each trial of the task, participants had to correctly discriminate between two stimuli of the target modality (visual or auditory) that were presented in rapid succession, while ignoring stimuli of the un-attended (or non-target) modality (Figure 2A). Each trial contained two successive stimuli of each modality that were presented for 500 ms each and were separated by a noise mask that lasted 500 ms. Participants judged whether the first and second stimulus (of the target modality) were identical or different. They were given 1500 ms to respond by pressing a button with their left or right index finger to indicate “same” or “different”. Visual stimuli presented on “different” trials varied in grating width (i.e., spatial frequency of the horizontal gratings), while visual stimuli presented on “same” trials had identical grating widths (i.e., identical spatial frequency of the horizontal gratings). Auditory stimuli presented on “same” trials had identical temporal frequencies, while auditory stimuli presented on “different” trials had different temporal frequencies. For the visual stimuli, the noise mask was a visual white noise pattern that was filtered to include spatial frequencies similar to the range of frequencies of the visual stimuli. For the auditory stimuli, the noise mask was auditory white noise that was filtered to include similar temporal frequencies to the range of auditory stimuli. A question mark replaced the fixation cross during the 2 s during which the participant could make a response. In order to standardize the difficulty of the task across participants, each participant’s just noticeable difference (JND) threshold for auditory and visual stimuli were measured prior to the scanning sessions. Thresholds were defined using the QUEST algorithm (Watson and Pelli, 1983) as the stimulus difference (in % difference between two stimuli) at which participants could correctly discriminate two sequentially presented 500 ms duration stimuli 70% of the time, and were measured independently for auditory and visual stimuli as previously described (Elkhetali et al., 2015). A schematic of the task design is shown in Figure 2A.

**Figure 1:**
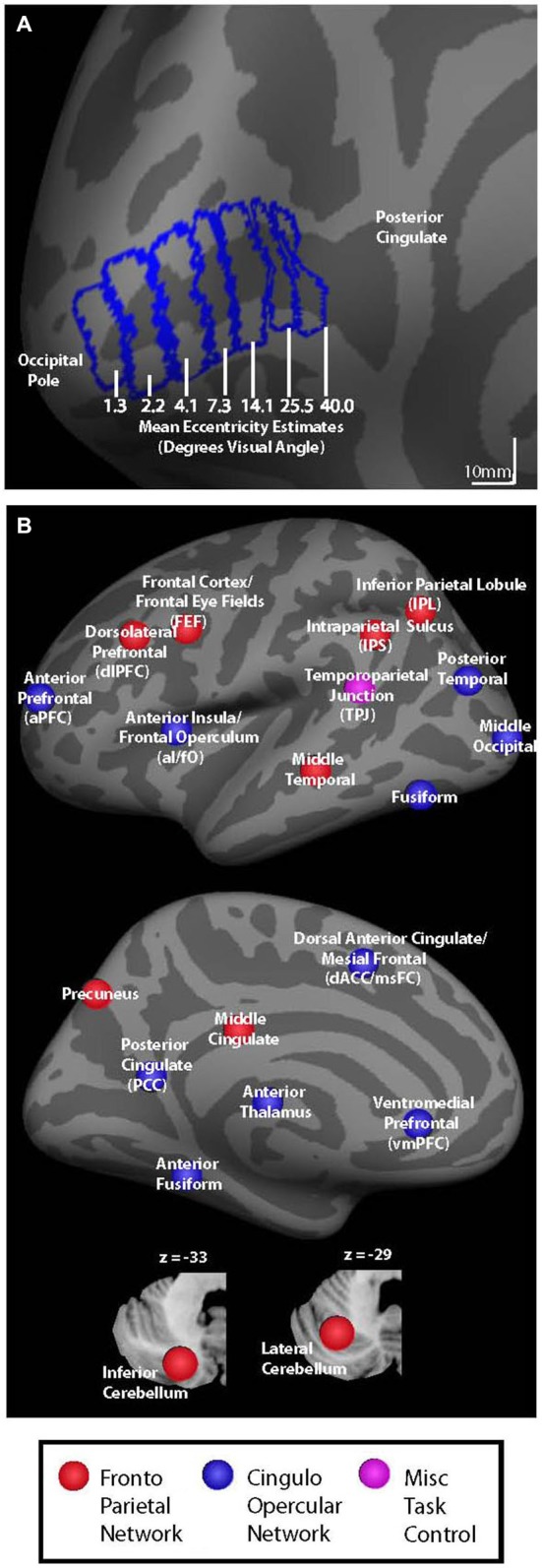
**(A)** V1 seed regions for background connectivity analyses are shown on the fsaverage inflated left hemisphere. Seed regions had mean eccentricity estimates of 1.3, 2.2, 4.1, 7.3, 14.1, 25.5, and 40.0° visual angle according to the retinotopy template developed by Benson et al. (2012). **(B)** Colored circles representing nodes associated with cingulo-opercular (blue), fronto-parietal (red), and miscellaneous (pink) task control networks are shown on an inflated left hemisphere brain and on cerebellar slices (bottom).

**Figure 2:**
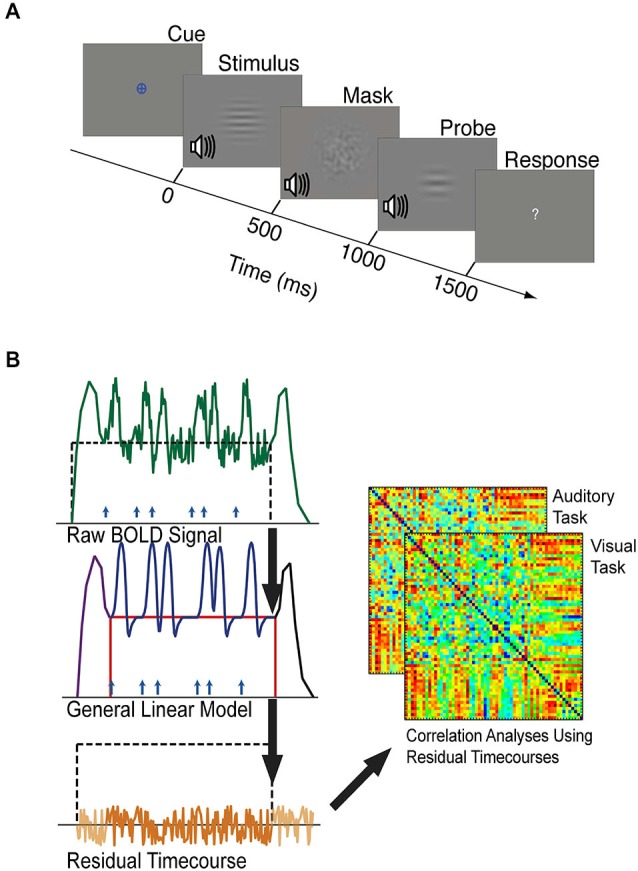
**(A)** The timeline of one trial of the visual or auditory attention task. Each task block was preceded by a period of resting fixation, and a colored cue indicated the target modality of each task block. Visual and auditory stimuli were presented at jittered intervals following the appearance of the colored cue. Stimuli, masks, and probes were presented for 500 ms each, and participants were given 1500 ms to respond with a button press indicating whether the stimulus and probe of the target modality were the same or different. Visual stimuli were considered different if they did not have matching spatial frequencies. Auditory stimuli were considered different if they did not have matching temporal frequencies. **(B)** Schematic outlining background connectivity analysis steps. A general linear model (GLM) was applied to the raw BOLD signal to account for variance associated with the onset of task blocks (purple), the offset of task blocks (black), individual trials (blue), and sustained shifts in baseline (red). The residual BOLD time-series was extracted from each seed region and region of interest. Correlations between these residual time-series were computed to estimate the background functional connectivity between each V1 eccentricity sector and each control network region of interest.

In this experiment, we were interested in testing whether or not the level of background connectivity between V1 and cognitive control networks is modulated in a retinotopically specific fashion by task factors. It is important to note that the auditory and visual attention tasks were chosen because they allowed identical stimulation with different tasks—the same stimuli were presented in both tasks, and only the target stimulus modality differed. The auditory task can therefore be thought of as a control condition—it contains identical stimuli and an active attentional component. Thus, changes in background connectivity between the visual and auditory tasks can be interpreted as being driven by task factors that are independent of the stimuli presented and simply performing a task.

### MRI Data Acquisition

Whole-brain BOLD-weighted images were obtained using a 3 T Siemens Allegra MRI scanner with a TR of 2 s, TE of 30 ms, and a voxel size 3.75 × 3.75 × 4 mm. At the beginning of each session, an anatomical MPRAGE scan was obtained of a participant’s brain, producing an image with a voxel size of 1.0 × 1.0 × 1.1 mm. Retinotopic localizer scans were performed during the first session, but were not used in defining the ROIs in the current study because each participant’s retinotopy was only mapped to approximately 8° visual angle and we expected that our ability to detect changes in the retinotopic patterns of background connectivity between V1 and control network nodes would be increased by also considering connectivity with more peripheral eccentricity sectors. Participants performed the tasks described above in alternating runs following an event-related design and the mixed blocked/event-related design described above. All data are described in another publication (Elkhetali et al., 2015), but for this analysis only data from the mixed blocked/event-related design were used. This design allowed us to use a general linear model to estimate components of fMRI activity associated with distinct aspects of the task, thus reducing the likelihood that results would be driven by externally driven correlations in activity (Visscher et al., 2003; Fair et al., 2007).

The visual stimuli were presented using a rear projection screen located outside of the magnet bore. The screen was visible through an angled mirror attached to the head coil that was placed above the participant’s eyes. The auditory stimuli were delivered to the first seven participants through MR safe Etymotics ER 30 earphones, with additional MR compatible ear protectors. However, due to participant discomfort with the in-ear devices, their use was substituted with auditory stimuli fed through the Siemens sound system via specialized headphones for the final 13 participants. There was no significant difference in performance between the two earphone models used.

### Preprocessing of fMRI Data

fMRI data were pre-processed using MATLAB scripts implementing pre-processing routines found in SPM8 (Friston et al., 1994). BOLD volumes were slice-time corrected, realigned and re-sliced, resampled to a 2 × 2 × 2 mm voxel size, and normalized to an EPI template (MNI space) using rigid body translation and rotation. The normalized BOLD data were then smoothed using a 5 mm FWHM Gaussian kernel. Motion-correction was applied using MATLAB scripts to minimize artifacts caused by movement. Motion correction was achieved by replacing volumes in which participants moved more than 0.5 mm in one TR (2 s) with an interpolated volume made from adjacent volumes. Runs were excluded if mean movement across the run was greater than 3 mm. Each participant included in the analysis had fMRI and behavioral data for at least nine blocks (72 trials) of each task. The average percentage of time-points excluded per participant was 7.05% with a standard deviation of 16%. The maximum number of excluded time-points was 38%.

### GLM Analyses

Task-driven neural responses were estimated from the BOLD volumes using SPM8, based on the general linear model approach (Friston et al., 1994). We modeled trial-driven effects, sustained task block effects, and task onset and offset effects (Figure 2B). Trial-driven effects were modeled with 12 regressors (1 per TR, corresponding to 24 s) in a finite impulse response (FIR) model, representing changes in activity that occurred in response to the stimulus. Task onset effects were modeled using seven FIR regressors (14 s) representing changes in activity that occurred after the start of the block. Task offset effects were modeled as 12 FIR regressors (24 s) representing changes in activity that occurred after the end of the block. Sustained task block effects were modeled using a single boxcar-shaped regressor that started 16 s from the beginning of the block (immediately following the task-initiation regressors) and ended at the end of a block. The regressor for task-maintenance activity was not convolved with any canonical hemodynamic response function because it represents a stable shift in baseline. More detailed descriptions of how the fMRI data were modeled can be found elsewhere (Visscher et al., 2003; Petersen and Dubis, 2012; Elkhetali et al., 2015).

### Pre-Processing for Background Connectivity Analyses

The residual (as in “residual error”) BOLD time-courses containing the signal variance that was not accounted for by our model (Figure 2B) were used to estimate background connectivity between V1 and control network nodes (Al-Aidroos et al., 2012). Additional pre-processing steps were performed on these residual time-courses to reduce spurious variance that was not related to neural activity (Fair et al., 2007). These steps were performed using MATLAB scripts implementing MATLAB functions to perform temporal band pass filtering (0.009 < *f* < 0.08 Hz), the regression of head motion parameters obtained during motion-artifact correction, the rejection of volumes containing greater than 0.5 mm of motion per TR, and the regression of the principal components of white matter and CSF signals. MATLAB scripts were also used to employ a motion scrubbing algorithm that was developed by Power et al. (2012) according to recommendations for ordering these steps to optimally control for motion-related artifacts (Carp, 2013). After these steps were completed, the whole-brain residual time-courses were extracted and concatenated into visual and auditory epochs. Correlations between these residual time-courses are referred to as “background connectivity” because they include correlations of moment-to-moment fluctuations, but do not include correlations driven by the average response to the trials or other modeled task factors (Al-Aidroos et al., 2012; Norman-Haignere et al., 2012).

### Regions of Interest Definition

V1 eccentricity sectors were defined manually on the Freesurfer fsaverage brain using the Freesurfer fsaverage V1 label file as a boundary. Starting at the most posterior vertex in the fsaverage V1 label file, we used the Freesurfer tool plot_curv to delineate consecutive strips of cortex that spanned ~10 mm from anterior to posterior (as shown in Figure 1A). Each V1 eccentricity sector was thus defined as a 10 mm anterior-posterior sector of the Freesurfer fsaverage V1 label file, resulting in nine total sectors. The most anterior and most posterior strips were discarded in order to prevent edge-of-brain effects at the occipital pole and to reduce the potential for including signal from other more anterior regions due to smoothing. It is important to note that V1 eccentricity sectors were only hand drawn for the fsaverage brain using the included V1 label file as a guide rather than being hand drawn for each subject individually. The V1 eccentricity sectors that were defined using the fsaverage V1 label file were then transformed to each subject’s space, and from each subject’s space to MNI space. All analyses were carried out in MNI space. For each eccentricity sector, mean eccentricity estimates were calculated using the Freesurfer retinotopy template developed by Benson et al. (2012). The seven eccentricity sectors defined based on strips of cortex in V1 had mean eccentricity estimates of 1.3, 2.2, 4.0, 7.3, 14.1, 25.5, and 40.0° visual angle and were used as seed regions for the background connectivity analyses, as shown in Figure 1A. While these eccentricity estimates are based of a probabilistic anatomical template, the location and retinotopy of V1 have been shown to be well predicted by the cortical anatomy (Hinds et al., 2008, 2009; Benson et al., 2012). Right and left hemisphere V1 seed regions were combined to create a single seed region for each eccentricity sector. The resulting V1 eccentricity sectors are shown on the left hemisphere fsaverage brain in Figure 1A. Our motivation to combine the seed regions from each hemisphere came partly from previous studies indicating that homologous eccentricity sectors in left and right hemispheric V1 are very strongly functionally connected (e.g., Raemaekers et al., 2014), and because the consistent central presentation of visual stimuli/fixation marks/cues did not give us reason to expect visual field-related differences in the background connectivity of left vs. right V1.

Thirty-seven additional ROIs were defined using the Marsbar toolbox for SPM.^1^ Each ROI was defined as a 5 mm radius sphere centered on co-ordinates that have been previously reported as key nodes of the cingulo-opercular and fronto-parietal control networks (Dosenbach et al., 2007). All ROIs were masked to include only in-brain voxels using the total intracranial volume binary mask included in SPM8. Control network nodes are shown in Figure 1B, and center co-ordinates and region names are given in Table 1. Regions in Table 1 are organized based on their network designation according to previous work (Dosenbach et al., 2007).

**Table 1:** **Control network ROI center coordinates**.

Regions of Interest	*x*	*y*	*z*
*Fronto-parietal network: primary*			
R IPS	30	−61	39
L IPS	−31	−59	42
R frontal cortex	41	3	36
L frontal cortex	−41	3	36
R precuneus	10	−69	39
L precuneus	−9	−72	37
Mid cingulate	0	−29	30
R IPL	51	−47	42
L IPL	−51	−51	36
R dIPFC	43	22	34
L dIPFC	−43	22	34
*Fronto-parietal network: auxiliary*			
R lat cerebellum	31	−61	−29
L lat cerebellum	−32	−66	−29
R inf cerebellum	18	−80	−33
L inf cerebellum	−19	−78	−33
R mid temporal	51	−33	−2
L mid temporal	−53	−31	−5
*Cingulo-opercular network: primary*			
R al/fO	36	16	4
L al/fO	−35	14	5
dACC/msFC	−1	10	46
R aPFC	27	50	23
L aPFC	−28	51	15
R ant thalamus	10	−15	8
L ant thalamus	−12	−15	7
*Cingulo-opercular network: auxiliary*			
R ant fusiform	25	−44	−12
L ant fusiform	−25	−44	−12
R fusiform	35	−65	−9
L fusiform	−34	−62	−15
R post temporal	44	−74	26
L post temporal	−40	−78	24
L mid occipital	−27	−89	3
R mid occipital	27	−89	3
R post cingulate	10	−56	16
L post cingulate	−11	−57	13
vmPFC	1	31	−2
*Miscellaneous*			
R TPJ	53	−46	17
L TPJ	−53	−46	17

### Background Connectivity Analyses

Subject-level background connectivity analyses consisted of first extracting the across-voxel average of the residual time-course for each task epoch from each control network region of interest and each V1 eccentricity sector region of interest using the Marsbar toolbox for SPM. Background connectivity between each V1 eccentricity sector and each control network node was then estimated by calculating the Pearson’s correlation coefficient between the extracted residual time-courses. The resulting first-level correlation maps were then transformed to Fisher z-score maps using Fisher’s r-to-z transform. For each control network node, group-level analyses consisted of entering the subject-level Fisher z-score values quantifying background connectivity between the control network node and the V1 eccentricity sectors into a two-way repeated measures ANOVA with within-subjects factors of eccentricity (seven levels, one for each V1 eccentricity sector seed) and target modality (two levels, one for each target modality). Because the purpose of this analysis was to identify control network nodes that showed changes in the retinotopic pattern of background connectivity with V1 between blocks of the auditory vs. visual target modalities, only results from control network nodes showing target modality-by-eccentricity interactions that were significant at a Family-Wise Error Rate (FWER) controlled *p* < 0.05 are reported. FWER for the ANOVAs was controlled using a Bonferroni correction.

Significant target modality-by-eccentricity interaction effects were followed up using Tukey’s Honestly Significant Difference (HSD) test to control the FWER across all pairwise comparisons between means. For each control network node that showed a significant target modality-by-eccentricity interaction, follow-up tests consisted of: (1) comparing background connectivity (Fisher z-score) between all pairs of V1 eccentricity sectors for both visual and auditory target modalities; (2) comparing background connectivity (Fisher z-score) for each V1 eccentricity sector between visual and auditory target modalities (Δ Fisher z-score); and (3) comparing Δ Fisher z-score for visual vs. auditory modalities between all pairs of V1 eccentricity sectors. The comparisons between pairs of V1 eccentricity sectors for a given condition (item 1 from the list above) were used to make inferences about the retinotopic pattern of background connectivity during attention to each target modality. The between-condition comparisons for each V1 eccentricity sector (item 2, Δ Fisher z-score) were used to make inferences about which V1 eccentricity sectors showed strong changes in background connectivity between visual vs. auditory target modalities. The comparison of the effect of modality between all pairs of V1 eccentricity sectors (item 3) were used to make inferences about how differences between pairs of eccentricity sectors changed between visual vs. auditory target modalities. Tukey’s HSD results were considered significant for *p* < 0.05.

### Retinotopic Connectivity Index (RCI) Definition

Because we were interested in how task-related changes in the retinotopic pattern of background connectivity between V1 and control network nodes related to task performance, we calculated a subject-level summary statistic that could be correlated with subject-level performance metrics. For each control network node showing a significant target modality-by-eccentricity interaction effect, subject-level differences in background connectivity between foveal (mean eccentricity estimate of 1.3° visual angle) and peripheral (mean eccentricity estimate of 40.0° visual angle) V1 eccentricity sectors were quantified by calculating a Retinotopic Connectivity Index (RCI) for each task. For each participant, the RCI between V1 and each control network node was defined as the difference in background connectivity (Δ Fisher z-score) between the most foveal V1 eccentricity sector (mean eccentricity estimate of 1.3° visual angle) and the most peripheral V1 eccentricity sector (mean eccentricity estimate of 40.0° visual angle). Thus, the RCI provided a subject-level index of the degree to which background connectivity with V1 differed between foveal and peripheral eccentricity sectors for visual and auditory target modalities. For each participant, the between-condition difference in RCI was used as an estimate of how the retinotopic pattern of background connectivity between V1 and each control network node was modulated by attentional processes. Thus, the difference between the RCI for the visual condition vs. the RCI for the auditory condition was used as a subject-level statistic reflecting how much the difference in the background connectivity of the most foveal and most peripheral V1 eccentricity sectors changed between blocks with auditory vs. visual target modalities. A positive RCI indicates that the background connectivity was higher for the foveal eccentricity sector, whereas a negative RCI indicates that background connectivity was higher for the foveal sector. For each control network node that showed a significant target modality-by-eccentricity interaction effect, linear correlation analyses (Pearson’s r) were performed to investigate whether, across participants, visual-auditory modulations of RCIs correlated with the total percent correct or average correct trial response times for the visual task.

It is worth noting that the RCI metric does not incorporate information about differences in background connectivity for every pair of V1 eccentricity sectors, and RCI modulations are not sufficient for making inferences about between-condition changes in the retinotopic pattern of background connectivity. However, a significant target modality-by-eccentricity interaction effect for background connectivity between V1 and a control network node indicates that the retinotopic pattern of background connectivity significantly differed between visual vs. auditory target modalities at the group level. Inferences about the specific changes underlying this effect should be based on the *post hoc* follow-up tests. Thus, while between-condition changes in RCI provide a single, subject-level metric that summarizes the retinotopic change in background connectivity between visual vs. auditory target modalities, specific inferences about these changes should be drawn from the results of the ANOVAs and follow-up tests.

## Results

### Behavioral Results

In general, participants performed well for both visual and auditory target modalities. The mean percent correct for the visual target modality was 81% with a standard deviation of 11%. The mean percent correct for the auditory target modality was 81% with a standard deviation of 12%. Mean percent correct did not differ significantly between target modalities (*t*_14_ = 0.06, *p* = 0.95), indicating that they were likely similar in difficulty. The mean reaction time for the visual target modality was 0.93 s with a standard deviation of 0.10 s. The mean reaction time for the auditory target modality was 1.11 s with a standard deviation of 0.15 s. Mean reaction time was significantly longer for the auditory target modality (*t*_14_ = −5.09, *p* < 0.001), and may reflect the fact that the auditory stimuli varied temporally, whereas the visual stimuli varied spatially. These results are consistent with the idea that the conditions are reasonably well equated despite their differences in modality (Visscher et al., 2007).

### Background Connectivity Results

**Figure 3:**
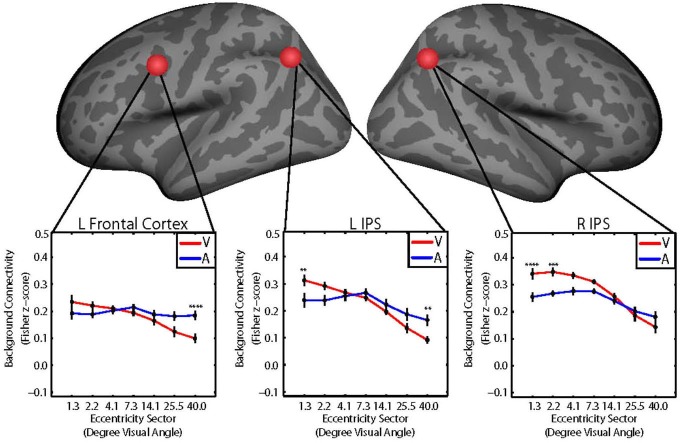
**Group average plots illustrating the retinotopic patterns of background connectivity (Fisher z-score) between V1 and control network nodes in the left frontal cortex (left), left intraparietal sulcus (IPS; middle), and right IPS (right) that showed significant target modality-by-eccentricity interactions for visual (red) and auditory (blue) target modalities**. Error bars represent within-subjects standard error. *Post hoc* tests were multiple comparisons corrected using Tukey’s honestly significant difference (HSD). V1 eccentricity sectors marked with asterisks showed significant changes in background connectivity with the control network node between visual and auditory target modalities. Note: *Post hoc* tests were performed using Tukey’s HSD to control Family-Wise Error Rate. *p < 0.05, **p < 0.01, ***p < 0.005, ****p < 0.001.

We hypothesized that the retinotopic patterns of background connectivity between V1 and control network nodes would differ between attention to visual vs. auditory target modalities. We performed two-way repeated measures ANOVAs with within-subjects factors of eccentricity (seven levels, one for each V1 eccentricity sector) and target modality (two levels, one for each target modality) for background connectivity measurements (Fisher z-score) between V1 and each of the 37 control network nodes to identify control network nodes showing significant target modality-by-eccentricity interaction effects on background connectivity with V1. Reported results are significant at *p* < 0.05, FWE-corrected using Bonferroni correction. These analyses revealed significant two-way interaction effects of target modality and eccentricity on background connectivity with V1 for the control network nodes with coordinates in the left frontal cortex near the FEF (*F*_6,84_ = 5.20; *p* = 0.005, FWE-corrected), the left IPS (*F*_6,84_ = 7.53; *p* = 0.0004, FWE-corrected), and the right IPS (*F*_6,84_ = 5.97; *p* = 0.001, FWE-corrected). Coordinates for the center of each control ROI are listed in Table 1. Group average plots illustrating background connectivity between each V1 eccentricity sector and each of these control network nodes are shown in Figure 3.

**Figure 4:**
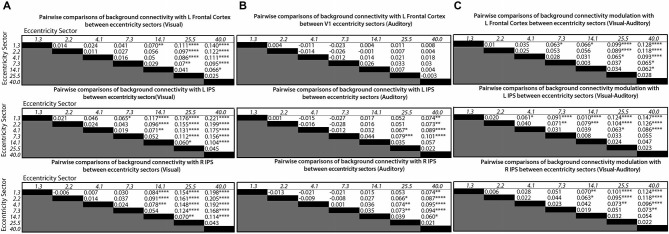
**Matrices showing differences in mean levels of background connectivity between all pairs of V1 eccentricity sectors and each of the three control network nodes identified by the ANOVA analyses for different conditions in (A), (B) and (C)**. The italicized numbers on the *y*-axes and *x*-axes correspond to the estimated mean eccentricity in visual angle for each V1 eccentricity sector. Each cell of the matrices in **(A)** contains the difference in the mean levels of background connectivity between the corresponding pair of V1 eccentricity sectors for the visual target modality (Mean Fisher z-score_row_−Mean Fisher z-score_column_), Each cell of the matrices in **(B)** contains the difference in the mean levels of background connectivity between the corresponding pair of V1 eccentricity sectors for the auditory target modality (Mean Fisher z-score_row_−Mean Fisher z-score_column_). Each cell of the matrices in **(C)** contains the difference between the values given in **(A)** and **(B)**. ([Mean Fisher z-score_row_−Mean Fisher z-score_column_]_visual_−[Mean Fisher z-score_row_−Mean Fisher z-score_column_]_auditory_). Positive values indicate that the difference between a pair of V1 eccentricity sectors was greater for the visual target modality than for the auditory target modality. Note: *Post hoc* tests were multiple comparisons corrected using Tukey’s HSD, *p < 0.05, **p < 0.01, ***p < 0.005, ****p < 0.001.

Follow-up tests for each control network node were performed using Tukey’s HSD to control FWER for all pairwise comparisons between means. V1 eccentricity sectors that showed significant (*p* < 0.05, FWE-corrected) differences in the level of background connectivity with each control network node between blocks with auditory vs. visual target modalities are marked with asterisks in Figure 3A. Pairs of V1 eccentricity sectors that significantly (*p* < 0.05, FWE-corrected) differed in background connectivity with each control network node for the visual target modality are marked with asterisks in Figure 4A. Pairs of V1 eccentricity sectors that significantly (*p* < 0.05, FWE-corrected) differed in background connectivity with each control network node for the auditory target modality are marked with asterisks in Figure 4B. Pairs of V1 eccentricity sectors whose differences in background connectivity significantly (*p* < 0.05, FWE-corrected) differed in background connectivity with each control network node between visual and auditory target modalities are shown in Figure 4C.

The follow-up tests revealed that background connectivity between V1 eccentricity sectors and the control network nodes showed a stronger retinotopic gradient during visual blocks than during auditory blocks (Figures 3A, 4C). Analyses of the left frontal cortex ROI revealed that while only the most peripheral (~40.0° visual angle) V1 eccentricity sector showed a significant change in background connectivity between visual and auditory target modalities (Figure 3A, left), significant differences between foveal and peripheral eccentricity sectors were only observed during visual blocks (Figure 4A, top; Figure 4B, top). In fact, the magnitudes of differences between foveal and peripheral V1 eccentricity sectors significantly differed between target modalities, with differences between foveal and peripheral eccentricity sectors being signifcantly larger for the visual target modality than for the auditory target modality (Figure 4C, top). Analyses of the left IPS ROI revealed that only the most foveal (~1.3° visual angle) and most peripheral (~40.0° visual angle) V1 eccentricity sectors showed a significant change in background connectivity between visual and auditory target modalities (Figure 3A, middle). Nonetheless, significant differences were observed between more eccentricity sectors for the visual target modality than for the auditory target modality (Figures 4A/B, middle). Differences between foveal and peripheral eccentricity sectors were significantly larger for the visual target modality than for the auditory target modality (Figure 4C, middle). Analyses of the right IPS ROI revealed that while only the first and second most foveal V1 eccentricity sectors (~1.3 and 2.2° visual angle, respectively) showed significant changes in background connectivity between auditory and visual target modalities (Figure 3A, right), significant differences were observed between more pairs of V1 eccentricity sectors for the visual target modality than for the auditory target modality (Figures 4A/B, bottom). Differences between foveal and peripheral eccentricity sectors were significantly larger for the visual target modality than for the auditory target modality (Figure 4C, bottom).

### RCI Behavioral Correlation Results

**Figure 5:**
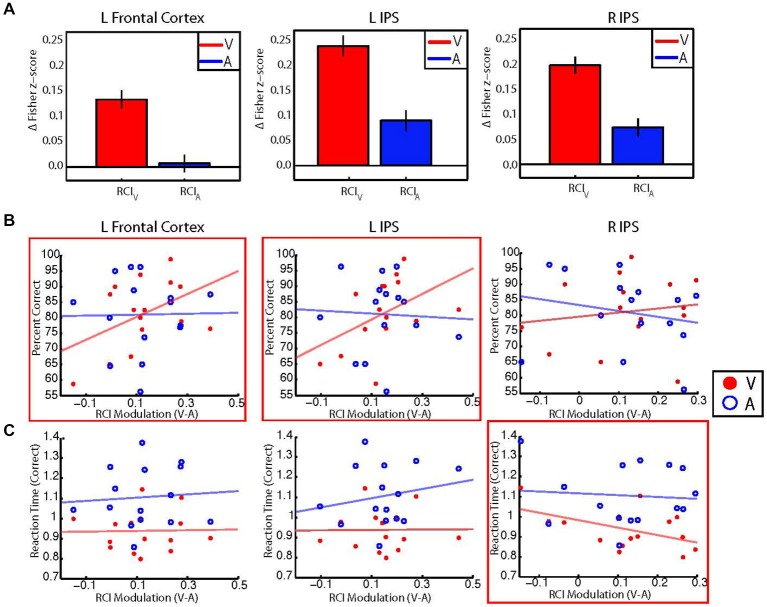
**(A)** Bar graphs showing group averaged retinotopic connectivity indices (RCIs) for visual (red) and auditory (blue) target modalities for each control network node. RCIs at all nodes were larger for the visual than auditory target modalities, indicating that mean differences between foveal and peripheral eccentricity sectors were greater during the visual task. Error bars represent within-subjects standard error. Note that information in **(A)** can also be observed by examining the most central and most peripheral ROIs in Figure 3. **(B)** Scatterplots illustrating the relationship between individual participant performance accuracy (% correct) and individual between-condition modulations of the RCI for each control network node. RCI modulations for the left frontal cortex (left) and left IPS (center) show moderate positive correlations with performance accuracy for the visual target modality (red), but not the auditory target modality (blue). No significant correlations were found between RCI modulations for the right IPS (right) and performance accuracy for either modality. **(C)** Scatterplots illustrating the relationship between correct trial response times and between-condition RCI modulations for each control network node. RCI modulations for the right IPS (right) show a moderate negative correlation with response times for correct responses on visual trials, but not for correct responses on auditory trials. No significant correlations were found between RCI modulations for the left frontal cortex or left IPS and correct trial response times for either modality. *Red boxes indicate significant (*p* < 0.05, uncorrected) correlations with performance for the visual target modality.

We reasoned that if changes in the retinotopic pattern of background connectivity between V1 and fronto-parietal areas reflected the selective enhancement of central visual processing by attention, then participants with larger modulations of RCI values between the auditory and visual conditions should also perform better on the visual task. Thus, for each control network node shown in Figure 3A, we investigated the relationship between performance accuracy on the visual task and between-condition RCI modulation. Visual and auditory RCI scores were calculated for each of the three control network nodes that showed significant target modality-by-eccentricity interaction effects as outlined in the Methods section. These RCI scores were correlated (Pearson’s r) with behavioral measures of reaction time and accuracy. Group-level average RCIs for visual and auditory blocks are shown for each ROI in Figure 5A.

Our *a priori* analyses revealed moderate positive correlations between performance accuracy for the visual target modality and RCI modulations in the left frontal cortex (*r* = 0.45, *p* = 0.04) and the left IPS (*r* = 0.46, *p* = 0.04), but did not reveal a significant relationship between RCI modulations and performance accuracy for the right IPS (*r* = 0.15, *p* = 0.29). Additional exploratory analyses revealed that performance accuracy on the auditory task did not correlate with RCI modulations for any of the three control network nodes (left frontal cortex: *r* = 0.01, *p* = 0.95; left IPS: *r* = −0.05, *p* = 0.86; right IPS: *r* = −0.20, *p* = 0.46). These results are in accordance with our hypothesis—participants with larger between-condition RCI modulations for the left frontal cortex and left IPS also performed more accurately for the visual target modality. Scatterplots illustrating the relationships between RCI modulation and percent correct for each modality are shown in Figure 5B.

Our *a priori* analyses did not reveal any significant correlations between correct trial response times for the visual target modality and RCI modulations for the left frontal cortex (*r* = 0.02, *p* = 0.53) or the left IPS (*r* = 0.01, *p* = 0.52), but did reveal a moderate negative correlation between correct trial response times for the visual target modality and RCI modulation for the right IPS (*r* = −0.49, *p* = 0.03). Additional exploratory analyses revealed that correct trial response times for the auditory target modality did not correlate with RCI modulations for any control network node (left frontal cortex: *r* = 0.07, *p* = 0.79; left IPS: *r* = 0.19, *p* = 0.48; right IPS: *r* = −0.08, *p* = 0.77). These results are also in accordance with our hypothesis—participants with larger between-condition RCI modulations for the right IPS also responded more quickly on correct trials of the visual task. Scatterplots illustrating the relationships between RCI modulation and correct trial response times for the visual and auditory tasks are shown in Figure 5C.

## Discussion

Processing information appropriately for a given task likely requires information to be routed in a precise, task-dependent manner. A growing body of evidence is consistent with this idea, and indicates that this routing of information may be accomplished by the configuration of task-specific patterns of connectivity among brain regions (Büchel and Friston, 1997; Friston and Büchel, 2000; Rowe et al., 2005; van Schouwenburg et al., 2010; Al-Aidroos et al., 2012; Norman-Haignere et al., 2012; Ebisch et al., 2013; Cole et al., 2014). Indeed, studies using background connectivity to characterize how connectivity among visual areas is modified by task factors have suggested that task-dependent changes in background connectivity may reflect the formation of task-dependent functional pathways for prioritized information transfer between brain regions (Al-Aidroos et al., 2012; Norman-Haignere et al., 2012). This interpretation is further supported by other studies that have found that retinotopic patterns of background connectivity within and between early visual areas are related to performance on visual attention tasks (Haynes et al., 2005), as well as by studies showing that background connectivity between early visual cortex and areas that are involved in attentional control depends on task factors (Ebisch et al., 2013). The data presented here demonstrate that task demands modulate the retinotopic gradient of background connectivity between V1 and regions in frontal and parietal cortex associated with attentional control, indicating that the attentional prioritization of visual information is accomplished in part by changes in the ongoing interactions between these regions and parts of early visual areas that correspond to task-relevant vs. task-irrelevant visual information. The finding that larger task-driven differences in the connectivity of these regions with foveal vs. peripheral V1 eccentricity sectors were associated with better visual task performance further supports this interpretation.

Our results show that when a central visual stimulus is attended, background connectivity between V1 and fronto-parietal areas becomes more highly differentiated across eccentricity sectors than when attention is directed instead to a simultaneous auditory stimulus (Figures 3A, 4). In this experiment, the visual stimuli were presented centrally in both conditions. While attention to the visual stimulus only resulted in strong between-condition changes in the background connectivity of one or two individual eccentricity sectors with fronto-parietal areas (Figure 3), the overall change in the retinotopic gradient of connectivity between V1 and these regions was much more pronounced (Figures 3, 4).

For the left frontal cortex, this gradient was driven primarily by decreased background connectivity to peripheral eccentricity sectors for the visual target modality compared to the auditory target modality, although small but statistically insignificant increases were observed for foveal eccentricity sectors as well (Figure 3A, left; Figure 4). Background connectivity with the left IPS showed a similar pattern—background connectivity to peripheral eccentricity sectors decreased for the visual target modality compared to the auditory target modality (Figure 3B, middle; Figure 4). The left IPS also showed increased background connectivity with the most foveal eccentricity sector for the visual target modality compared to the auditory target modality (Figure 3A, middle; Figure 4). The right IPS showed a somewhat different pattern background—connectivity with foveal eccentricity sectors increased for the visual target modality compared to the auditory target modality, but background connectivity with peripheral sectors did not differ greatly between target modalities (Figure 3A, right; Figure 4). These data indicate that the attentional prioritization of visual information is accomplished by the development of graded retinotopic differentiations in connectivity between early visual areas and fronto-parietal areas.

All three regions that showed changes in retinotopic connectivity patterns with V1 were primary nodes of the fronto-parietal control network (Dosenbach et al., 2007). The fronto-parietal control network is a distributed cortical network composed of regions that are involved in the goal-directed adjustment of sensory processing during task performance (Dosenbach et al., 2007; Ptak, 2012). Regions associated with the fronto-parietal network, particularly the FEF and IPS, have been strongly implicated in the top-down control of visual processing in humans (Hopfinger et al., 2000; Giesbrecht et al., 2003; Grent-’t-Jong and Woldorff, 2007; He et al., 2007; Kastner et al., 2007; Bressler et al., 2008; Szczepanski et al., 2010, 2014; Zanto et al., 2010, 2011; McMains and Kastner, 2011; Simpson et al., 2011; Greenberg et al., 2012; Nelissen et al., 2013). Our results are consistent with evidence that attentional modulations of neural activity in parts of early visual cortex that correspond to attended and unattended spatial locations likely result from top-down signals originating in the FEF and IPS (Bressler et al., 2008; Sylvester et al., 2008; Szczepanski et al., 2010; Simpson et al., 2011). Importantly, neural activity in these regions has previously been shown to predict activity in early visual cortex prior to the presentation of an expected visual stimulus (Bressler et al., 2008; Vossel et al., 2012). Similarly, a recent MEG experiment found that cue-related information results in a flow of activation from early visual cortex to parietal cortex to frontal cortex that is then followed by modulations of activity in early visual areas (Simpson et al., 2011). Studies of non-human primates further indicate that neurons in V1 and the FEF engage in reciprocal interactions during the selection and processing of task-relevant visual information (Pooresmaeili et al., 2014). While both the FEF and IPS clearly play very important roles in the control of visual processing, it has been suggested that the role of the IPS is to directly enhance neuronal activity within task-relevant areas of visual cortex, whereas the FEF may exert more indirect influences on neural activity in visual cortex (Liu et al., 2014). This might be expected, given that there is evidence for direct projections from the IPS to early visual areas V1–V3 in humans (Greenberg et al., 2012). However, evidence from TMS studies indicates that while both the FEF and IPS can directly modulate activity in early visual areas, these regions may serve different functions, and further suggests that the left vs. right hemispheric FEF and IPS may play distinct roles in the control of early visual processing. For example, while TMS of both the left FEF and right FEF has been found to reduce BOLD activity in central eccentricity sectors of V1, TMS of the right FEF has been found to also increase BOLD activity in peripheral eccentricity sectors of V1 (Ruff et al., 2009). Similarly, whereas stimulation of the right IPS has been found to increase BOLD activity in V1 when visual stimuli are absent, TMS of the left IPS has not been found to alter BOLD activity in V1 (Ruff et al., 2009). Although at first glance, our findings may appear to conflict with the findings from these TMS studies, it is important to note that the effects of TMS do not necessarily reflect the effects of attention during task performance. Our study also does not purport to show that the IPS and FEF are directly modulating BOLD activity in different eccentricity sectors of V1, but rather shows that the correlation of background activity between these regions and different eccentricity sectors in V1 depends on attentional factors. It is also worth noting that although the results from TMS studies suggest that interactions between V1 and the FEF/IPS are biased towards specific eccentricity sectors when no task is being performed, our data indicate that any retinotopic biases in the functional interactions between V1 and these regions are strongly influenced by attention even under identical stimulus conditions. This suggests that while certain regions may show “preferential” connectivity with specific eccentricity sectors in V1 when no task is being performed, these patterns of connectivity are likely malleable and can change according to task demands. Thus, our results integrate with previous studies to provide an important insight into how background interactions between V1 and the FEF and IPS reflect the allocation of visual attention.

There is growing evidence that the functional connectivity of the visual system strongly corresponds to retinotopic divisions (Buckner and Yeo, 2014; Raemaekers et al., 2014), and it is conceivable that attention-driven modulations of cortical state in V1 might be propagated to higher levels of the visual system to set up prioritized channels of information flow. This explanation is consistent with reports of task-dependent changes in background connectivity that occur between early visual areas and later visual areas that are involved in the specialized processing of faces and scenes (Al-Aidroos et al., 2012), and our results provide evidence that similarly prioritized channels may be configured between early visual and control network nodes in frontal and parietal cortex during the deployment of attention. This is in accordance with evidence that frontal and parietal cortices maintain topographic priority maps of visual space during various tasks involving attention to visual information that likely encode attentional biases to specific stimulus features and locations based on their behavioral relevance (e.g., task goals; Kastner et al., 2007; Jerde et al., 2012; Ptak, 2012; Klink et al., 2014). While speculative, it is possible that during task performance, heightened coupling between fronto-parietal areas and sectors of early visual areas that correspond to attended locations and features could in part lead to the emergence of topographic priority maps in frontal and parietal cortex.

In summary, we found that attention to visual stimuli influences the strength of background connectivity between V1 and control regions in frontal and parietal cortex. Further, our results indicate that these changes in connectivity depend on task demands rather than stimulus features, and differ for parts V1 that correspond to different eccentricity sectors. Rather than simply resulting in focal increases in background connectivity between fronto-paritetal areas and task-relevant eccentricity sectors, attention to a central visual target resulted in the development of a more highly retinotopically differentiated connectivity patterns than attention to a simultaneously presented auditory target. While further studies are necessary to fully characterize these effects within the context of more complex visual attention paradigms (e.g., paradigms including the presence of visual distracters at different eccentricities and/or in different hemifields), these results provide important insights into the cortical dynamics that enable the attentional prioritization of visual information. Rather than inducing large focal increases in the coupling of the FEF/IPS with eccentricity sectors that correspond to attended visual information, attention may result in the development of graded connectivity patterns that reflect top-down prioritizations of visual information at certain spatial locations.

## Conflict of Interest Statement

The authors declare that the research was conducted in the absence of any commercial or financial relationships that could be construed as a potential conflict of interest.

## Footnotes

^1^www.marsbar.sourceforge.net
